# *Toxoplasma gondii* PPM3H regulates the parasite virulence and modulates host immune and inflammatory responses in mice

**DOI:** 10.1186/s13567-025-01603-y

**Published:** 2025-08-26

**Authors:** Jun-Jun He, Jun Ma, Meng-Ling Deng, Hany M. Elsheikha, Yi-Dan Wang, Yu-Cong Zhang, Feng-Cai Zou, Xing-Quan Zhu

**Affiliations:** 1 The Yunnan Key Laboratory of Veterinary Etiological Biology, College of Veterinary Medicine, Yunnan Agricultural University, Kunming, 650201 Yunnan China; 2 State Key Laboratory for Animal Disease Control and Prevention, Key Laboratory of Veterinary Parasitology of Gansu Province, Lanzhou Veterinary Research Institute, Chinese Academy of Agricultural Sciences, Lanzhou, Gansu, 730046 China; 3 Faculty of Medicine and Health Sciences, School of Veterinary Medicine and Science, University of Nottingham, Sutton Bonington Campus, Loughborough, LE12 5RD UK; 4 Laboratory of Parasitic Diseases, College of Veterinary Medicine, Shanxi Agricultural University, Taigu, 030801 Shanxi China

**Keywords:** *Toxoplasma gondii*, PPM3H, PP2C phosphatases, parasite effector proteins, virulence, host–pathogen interaction

## Abstract

*Toxoplasma gondii* is an obligate intracellular parasite that causes severe illness in infants infected during pregnancy and in immunocompromised individuals. This parasite manipulates host cells through effector proteins that promote its survival and replication. While the phosphatases in the PP2C family have been shown to regulate host immune responses and contribute to the virulence and pathogenicity of various pathogens, the specific biological functions of PPM3H in *T. gondii* and its role in host–pathogen interactions remain unclear. In this study, we demonstrate that knockout of *ppm3h* significantly reduces the virulence and pathogenicity of *T. gondii*. In contrast, that high expression of *ppm3h* in the less virulent PRU induced by replacing the *ppm3h* gene elements of RH strain can enhance its pathogenicity, indicating a direct contribution of PPM3H to virulence in expression-independent manner. Furthermore, PPM3H significantly influenced host gene expression, with differentially expressed genes predominantly enriched in immune and inflammatory pathways. Weighted gene co-expression network analysis identified host immune genes, including chemokines such as *Cx3cl1* and *Ccl22*, as co-expressed with *ppm3h*. Also, *ppm3h* co-expressed with *T. gondii* rhoptry genes including *rop18*, a well-known virulence factor, suggesting a role for PPM3H in coordinating host–pathogen interactions. Our findings establish that PPM3H enhances *T. gondii* virulence by modulating the host immune and inflammatory responses. PPM3H does not impact parasite gene expression, invasion or replication in vitro, supporting its role as an immune modulator rather than a general fitness factor. This suggests that *T. gondii*’s pathogenicity arises not only from immune evasion but also from the active induction of host immune and inflammatory responses mediated by PPM3H.

**Supplementary Information:**

The online version contains supplementary material available at 10.1186/s13567-025-01603-y.

## Introduction

*Toxoplasma gondii* is a globally distributed opportunistic pathogen capable of infecting nearly all warm-blooded animals. Approximately one-third of the human population has been seropositive for *T. gondii* infection [1]. However, seroprevalence varies significantly by region, with higher rates reported in parts of Africa, Latin American, Europe and South America [2]. The widespread distribution of zoonotic *T. gondii* is facilitated by its unique survival strategies, enabling it to overcome complex cellular barriers, such as the placental and blood–brain barriers. This can result in severe complications including brain lesions, stillbirth, and neurological anomalies in congenitally infected infants [3]. Although approximately 75% of congenital cases are subclinical, surviving infants may experience long-term consequences, such as ocular damage, intracranial calcifications, and cognitive impairments [4]. In immunocompetent hosts, *T. gondii* establishes a lifelong chronic infection that is typically asymptomatic. However, in susceptible hosts, such as mice or immunocompromised patients (e.g., those with HIV), infection can be fatal.

*Toxoplasma gondii* is broadly classified into three major clonal lineages (type I, II, and III), distinguished primarily by their virulence in mice. Multiple virulence factors contribute to its pathogenicity, including secreted kinases [5]. For example, rhoptry protein 16 (ROP16) from type I strains is a kinase that phosphorylates the host transcription factor STAT3, activating the STAT3 pathway and suppressing IL-12 production [6]. IL-12 is critical for inducing IFN-γ production [7], which activates immune defense factors such as indoleamine 2,3-dioxygenase (IDO), inducible nitric oxide synthase (iNOS), immunity-related GTPases (IRGs), and guanylate-binding proteins (GBPs). IDO restricts *T. gondii* replication by degrading tryptophan [8], while nitric oxide produced by iNOS exhibits microbicidal activity [9]. IRGs and GBPs disrupt the parasitophorous vacuole membrane (PVM) that shelters *T. gondii* within host cells [7, 10–12]. However, rhoptry protein 18 (ROP18) from type I strains phosphorylates IRGs, preventing their targeting of the PVM and thereby preserving the vacuole’s integrity [13].

The survival and virulence of *T. gondii* depend heavily on the regulation of host and parasite protein phosphorylation, governed by a balance between kinases and phosphatases. Kinases add phosphate groups, while phosphatases remove them. A recent phosphoproteomics study revealed that nearly half of the proteins in eukaryotic organisms are phosphoproteins [14]. In *T. gondii*, phosphorylation regulates processes including bradyzoite differentiation, gene expression, cell cycle progression, motility, invasion, and egress [15, 16]. Therefore, elucidating the biological roles of *T. gondii* phosphatases is essential for understanding its pathogenic mechanisms. Among these, the PPM/PP2C family accounts for 44% of *T. gondii* protein phosphatases, according the ToxoDB/VEuPathDB database [17], highlighting their importance roles in the parasite’s lifecycle [18].

Phosphatases in pathogens often serve as key virulence factors. For example, phosphatases in *Francisella* modulate macrophage activity to facilitate survival and pathogenesis [19], while the bacterial-like phosphatase PfShelph2 in *Plasmodium* contributes to host cell invasion [20]. The PP2C phosphatase in *Leishmania* regulates host cytokines including IL-1β, IL-12P70 and IL-10 [21]. In *T. gondii*, 52 serine/threonine protein phosphatases have been identified, with most belonging to the PPM subfamily [16]. However, only a few PPM phosphatases have been characterized, and even fewer have been linked to virulence [22–24]. For example, PP2C-hn is a nuclear-localized phosphatase whose knockout causes mild growth defects [22]. PPM5C affects parasite attachment to host cells but not replication or egress [24]. PPM3C regulates the export of effector proteins (e.g., GRA16 and GRA28) from the vacuole lumen into the host cell [23]. According to the ToxoDB data and previous work [25], both PPM3C and PPM3H (GeneID: TGME49_201630) localize to the rhoptry, a critical organelle involved in cell invasion and immune evasion [23].

Previous research has mainly focused on PP2C-hn, PPM5C, and PPM3C, leaving the biological functions of PPM3H in host–pathogen interactions largely unexplored. The mechanisms by which PPM3H regulates *T. gondii* virulence and modulates host responses remain unclear. In this study, we used gene-editing and transcriptomic approaches to investigate the role of PPM3H in *T. gondii*. The study findings provide new insights into the parasite’s virulence mechanisms and its impact on host immune responses.

## Materials and methods

### Parasite strains and culture conditions

Tachyzoites of *T. gondii* strains RH and PRU were maintained through serial passage in human fibroblast foreskin (HFF) cells (ATCC®, Manassas, VA, USA). HFF cells were cultured in 25 cm^2^ plastic flasks using Dulbecco’s Modified Eagle’s Medium (DMEM) supplemented with 10% fetal bovine serum (FBS), 100 U/mL penicillin, and 100 μg/mL streptomycin. Cultures were incubated at 37 °C in a humidified incubator containing 5% CO_2._

### CRISPR/Cas9-mediated knockout and replacement of the *ppm3h* gene in *T. gondii* strains

CRISPR/Cas9 technology was employed to construct two genetically modified *T. gondii* strains: RH-PPM3H-KO, in which the *ppm3h* gene was knocked out in the RH strain, and PRU-PPM3H-R, in which the *ppm3h* gene in the PRU strain was replaced with a partial promoter, complete coding sequence (CDS), and 3' untranslated region (UTR) derived from the RH strain. These modified strains were generated to examine the functional role of PPM3H, investigate expression and regulation differences between strains, and evaluate whether PPM3H contributes to virulence independently of general parasite viability.

The RH-PPM3H-KO strain served as a loss-of-function model to assess whether PPM3H is essential for parasite viability, replication, or virulence in the RH background. In contrast, the PRU-PPM3H-R strain was designed to (1) determine whether regulatory or structural differences in the *ppm3h* locus between RH and PRU strains contribute to phenotypic variation, (2) evaluate whether RH-derived PPM3H can alter parasite behavior or virulence in the less virulent PRU background, and (3) provide a complementary approach to gene knockout, distinguishing between potential pleiotropic effects and direct PPM3H-specific functions.

Single-guide RNAs (sgRNAs) were designed using the E-CRISP tool, selecting sequences with no predicted off-target effects. These sgRNAs, targeting regions upstream or downstream of the *ppm3h* gene, were cloned into a CRISPR/Cas9 plasmid using the Q5 Site-Directed Mutagenesis Kit (New England Biolabs). The plasmid was kindly provided by Shen and colleagues [26]. For positive clone selection, a dihydrofolate reductase (DHFR) resistance cassette, conferring pyrimethamine resistance, was included.

**Figure 1:**
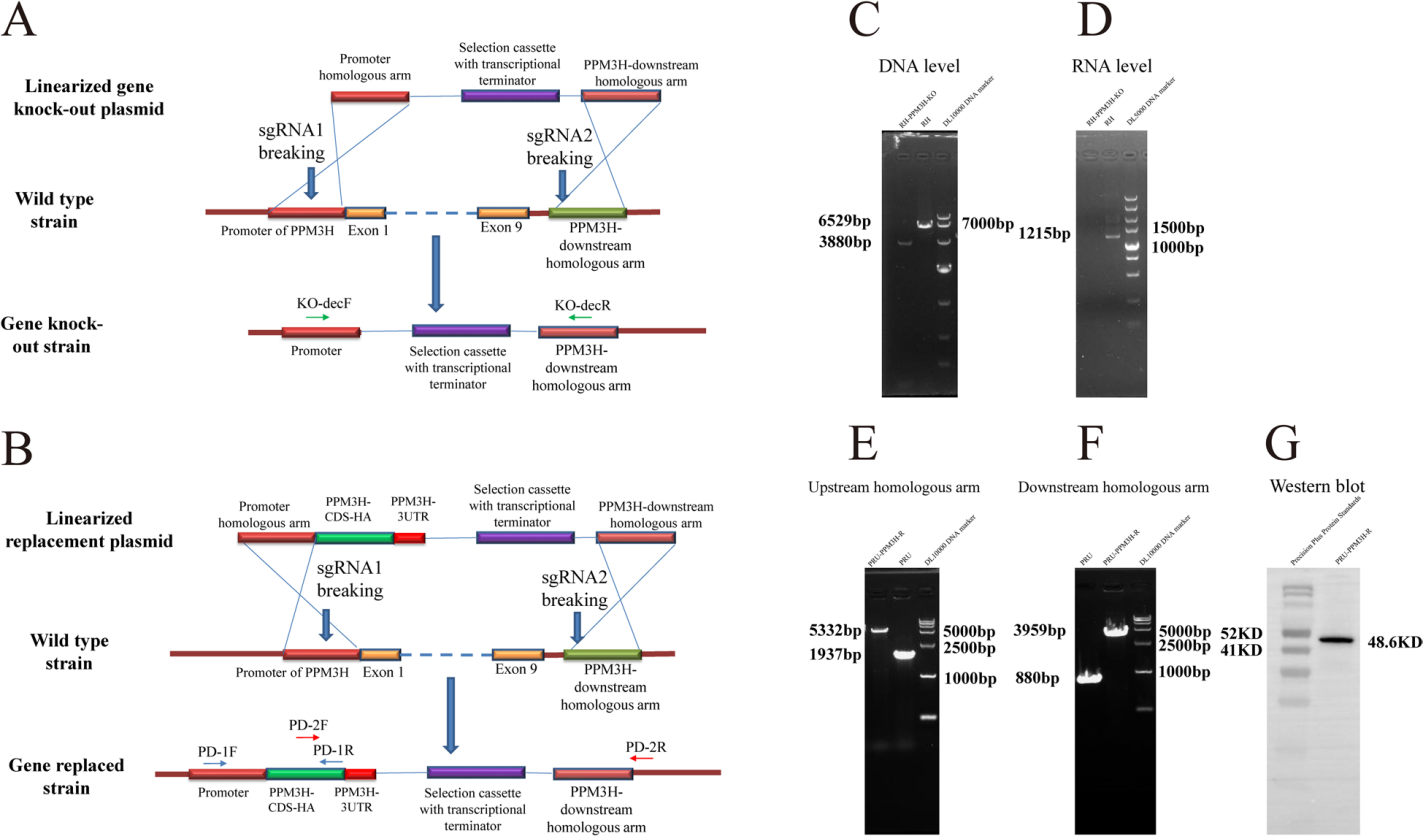
**Schematic and molecular validation of* ppm3h*** **gene editing in *Toxoplasma gondii***.** A** Schematic representation of the *ppm3h* knock-out strategy in the RH strain. The gene was disrupted using CRISPR/Cas9-mediated double-stranded breaks at two sgRNA-targeted sites flanking the coding sequence, replaced by a selection cassette under the control of the native promoter. **B** Schematic representation of *ppm3h* gene replacement in the PRU strain. A tagged version of *ppm3h* (CDS-HA) under its native promoter was reintroduced at the endogenous locus using homologous recombination. **C** PCR validation of genomic DNA confirming the deletion of *ppm3h* in RH-PPM3H-KO parasites. Amplicon sizes indicate successful gene disruption. **D** RT-PCR verification of *ppm3h* transcript absence in RH-PPM3H-KO parasites compared to wild-type RH parasites, indicating transcriptional silencing. **E–F** Genomic PCR confirmation of 5' and 3' homologous arm integration, validating correct insertion of the repair construct in the PRU-PPM3H-R strain. **G** Western blot detection of PPM3H-HA protein expression in PRU-PPM3H-R, confirming successful protein re-expression. Molecular weight markers are shown, and PPM3H-HA is detected at the shown size.

The construction of the knockout plasmid is illustrated in Figure 1A. The upstream homologous arm was amplified from RH strain genomic DNA using primers PPM3Hko-UF and PPM3Hko-UR. The DHFR selection cassette was amplified using primers DHFR-koF and DHFR-koR, and the downstream homologous arm using PPM3Hko-DF and PPM3Hko-DR. A linearized plasmid backbone was amplified from the PMD-19 T vector (Takara, China) using primers 19T-F and 19T-R. These PCR products were assembled using the ClonExpress MultiS One Step Cloning Kit (Vazyme, China).

To prevent sgRNA-mediated plasmid cleavage, point mutations were introduced into the homologous arms in both knockout and replacement constructs using the Mut Express^®^ MultiS Fast Mutagenesis Kit V2 (Vazyme, China). The final knock-out plasmid was linearized with the *NotI* restriction enzyme. Two sgRNAs targeting the *ppm3h* locus were co-transfected with the linearized plasmid into wild-type RH parasites, as previously described [26]. Forty-eight hours post-transfection, pyrimethamine selection was applied to isolate RH-PPM3H-KO clones. Successful gene disruption was confirmed at the DNA level using primers KO-decF and KO-decR, and at the RNA level using primers PPM3H-CDS-F and PPM3H-CDS-R. This approach enabled assessment of the consequences of *ppm3h* deletion in the highly virulent RH strain, offering insights into its role in parasite replication, survival, and gene regulation. The RH strain’s rapid growth and lack of cyst formation make it a suitable model for essential gene function analysis.

For the PRU-PPM3H-R strain, the gene replacement construct (Figure 1B) was assembled by amplifying the upstream homologous arm from RH genomic DNA using primers PPM3HR-UF and PPM3HR-UR. The HA-tagged PPM3H CDS was amplified from RH cDNA using PPM3H-HA-F and PPM3H-HA-R, and the 3′UTR using PPM3HR-3urt-F and PPM3HR-3utr-R. The DHFR selection cassette and downstream homologous arm were amplified using primers DHFR-F/DHFR-R and PPM3HR-DF/PPM3HR-DR, respectively. The vector backbone was amplified using primers 19T-F and 19T-R and assembled using the ClonExpress MultiS One Step Cloning Kit (Vazyme, China). The final plasmid was linearized with NotI.

The same two sgRNAs used in the knockout experiment were co-transfected with the linearized replacement construct into wild-type PRU parasites. Pyrimethamine selection was applied 48 h after transfection to isolate PRU-PPM3H-R clones. Successful gene replacement was confirmed using primers PD-1F, PD-1R, PD-2F, and PD-2R, and PPM3H expression in PRU-PPM3H-R parasites was validated via western blot.

This replacement strategy enabled a targeted investigation of whether differences in promoter, CDS, or 3′UTR regions between RH and PRU strains influence *ppm3h* expression or function. The PRU background was specifically chosen to determine whether RH-derived PPM3H enhances virulence in a strain with lower intrinsic pathogenicity. Furthermore, this approach complements the knockout model by avoiding confounding effects of gene deletion, enabling clearer interpretation of phenotypic changes associated with PPM3H activity. Given that PRU is a type II strain capable of cyst formation and exhibits distinct regulatory features, PRU-PPM3H-R is especially valuable for studying strain-specific regulation and functional divergence at the *ppm3h* locus.

### Survival and immune response in mice infected with *T. gondii* strains and *PPM3H* knockout mutants

Six-week-old female BALB/C specific pathogen-free mice were housed under pathogen-free conditions with ad libitum access to sterilized food and water. Following a one-week acclimatization period, the mice were randomly assigned to seven groups (*n* = 10 per group): a non-infected control group, an RH-infected group (RH group), an RH-PPM3H- knockout-infected group (RH-PPM3H-KO group), a PRU-infected group (PRU), and three PRU-PPM3H-R-infected groups that received varying doses of tachyzoites. Mice in the RH and RH-PPM3H-KO groups were intraperitoneally (i.p.) inoculated with 200 tachyzoites of the RH or RH-PPM3H-KO strain, respectively. The PRU group received 200 tachyzoites of the PRU strain via i.p. injection. The PRU-PPM3H-R-infected groups were challenged i.p. with 200, 500, or 2000 tachyzoites of the PRU-PPM3H-R strain. Control mice received mock injections of sterilized phosphate-buffered saline (PBS) via the same route. All mice were monitored daily to assess survival and humane end points. Serum cytokine levels were analyzed in nine surviving mice challenged with 200 tachyzoites from either the PRU or PRU-PPM3H-R strain, using the BD Cytometric Bead Array (CBA) Mouse Th1/Th2/Th17 Cytokine Kit (BD Bioscience, USA).

### Parasite invasion assay

Parasite invasion assays for the RH and RH-PPM3H-KO strains of *T. gondii* were performed as previously described [27]. Freshly egressed tachyzoites were harvested and resuspended in DMEM supplemented with 2% FBS. The tachyzoites were then added to confluent monolayers of HFF cells and incubated for a 20 min to allow parasite invasion. Following incubation, the monolayers were washed and fixed with 4% paraformaldehyde (PFA) in PBS for 20 min. Extracellular parasites were stained with a fluorescein isothiocyanate (FITC)-conjugated anti-*T. gondii* antibody (Invitrogen, USA). After permeabilization with 0.2% Triton X-100 for 15 min, the total parasite population (both intracellular and extracellular) was stained with a rabbit polyclonal anti-*T. gondii* antibody, followed by detection with a donkey anti-rabbit IgG antibody conjugated to Alexa Fluor® 568 (Abcam, USA). Parasites were quantified by counting five randomly selected fields per sample, across three independent biological replicates. Results were displayed as the percentage of intracellular parasites relative to the total parasite population and are presented as means ± standard error of the mean (SEM) from three independent experiments.

### Parasite replication assay

Parasite replication assays for the RH and RH-PPM3H-KO strains of *T. gondii* were performed as previously described [27]. Briefly, parasites were cultured in confluent HFF monolayers. At 36 h post-infection, the monolayers were fixed using 4% PFA in PBS and permeabilized with 0.2% Triton X-100 for 15 min. The total parasite population was stained using an FITC-conjugated anti-*T. gondii* antibody (Invitrogen, USA). The number of parasites per parasitophorous vacuole (PV) was counted in at least 50 PVs across 3 biological replicates. Results are presented as the means ± SEM from three independent experiments.

### Transcriptomic analysis of RAW264.7 cells challenged with* T. gondii*

RAW264.7 murine macrophage cells were cultured in T25 flasks using DMEM/F12 medium supplemented with 10% FBS, 100 Ug/mL streptomycin and 100 U/mL penicillin. Five experimental groups were established, including an RH group (infected with the RH strain), a PRU group (infected with the PRU strain), an RH-PPM3H-KO group (infected with the RH-PPM3H-KO strain), a PRU-PPM3H-R group (infected with the PRU-PPM3H-R strain) and a non-infected control group. Each group consisted of three biological replicates. Cells in the infected groups were challenged with the respective *T. gondii* strains at a multiplicity of infection (MOI) of 1 parasite per host cell. The control group received a mock treatment with an equal volume of PBS. At 24 h post-infection, cells from all five groups were harvested for RNA sequencing (RNA-seq).

Total RNA was extracted by using TRIzol Reagent (Invitrogen) following the manufacturer′s instructions. RNA integrity and concentration were assessed using an Agilent 2100 Bioanalyzer and a Nanodrop 2000, respectively. mRNA libraries were prepared using the IlluminaTruSeqTM RNA Sample Preparation Kit and sequenced on the IlluminaHiSeq2000 platform. Raw sequencing reads were quality-filtered to remove adaptors and low-quality sequences using fastp software [28]. Clean reads were aligned to the mouse genome (Ensembl database: GRCm39 version) and the *T. gondii* genome (ToxoDB database: ToxoDB-60_TgondiiME49 version) using the HISAT2 package in R.

Gene expression levels were quantified using the StringTie package in R. Differentially expressed genes (DEGs) were identified using the DESeq2 package with the following thresholds: Benjamini & Hochberg-adjusted *P*-value (padj) < 0.01 and |Log_2_(Fold Change)|> 1. Functional enrichment analyses, including Kyoto Encyclopedia of Genes and Genome (KEGG) pathway and Gene Ontology (GO) enrichment, were performed using KOBAS with a corrected *P*-value < 0.01 as the significance threshold.

Weighted Gene Co-Expression Network Analysis (WGCNA) was conducted in R following the standard protocol. Co-expression modules were identified using a significance threshold of *P* ≤ 0.05. Genes within significant co-expression modules and with gene significance *P* < 0.01 were considered co-expressed with *T. gondii ppm3h*.

### Quantitative real-time PCR (qPCR) validation of DEGs

To validate the gene expression data, 15 DEGs were randomly selected for analysis using qPCR. Total RNA was reverse-transcribed into cDNA using M-MLV reverse transcriptase (Promega, Beijing, China) according to the manufacturer’s instructions. *Gapdh* was used as the endogenous reference gene for normalization of the qPCR data. qPCR was performed using the Rotor-Gene Q system (QIAGEN, Hilden, Germany) with SYBR Green GoTaq qPCR Master Mix (Promega, Beijing, China), according to the manufacturer’s protocol. The cycling conditions were as follows: initial denaturation at 95 °C for 5 min, followed by 40 cycles of 95 °C for 10 s, 60 °C for 15 s, and 72 °C for 20 s. A melt curve analysis was conducted from 72 °C to 95 °C to verify amplification specificity. Relative gene expression changes were calculated by using the 2^−ΔΔCT^ method [29].

### Primers and statistical analysis

All primers used in this study are listed in Additional file 1. Statistical analyses were performed using R (version 4.1) and GraphPad Prism 9 software. Comparisons between two groups were conducted using two-tailed Student’s *t*-tests. Data are presented as mean ± SEM, with error bars representing SEM.

## Results

### Impact of *ppm3h* gene editing on *T. gondii* virulence, invasion, and replication

As shown in Figure 1C, PCR analysis using primers KO-decF and KO-decR produced a 6529 bp fragment in the wild-type RH strain, while a smaller 3880 bp fragment was observed in the RH-PPM3H-KO strain, confirming successful deletion of *ppm3h*. This was further supported at the transcript level, where RT-PCR using PPM3H-CDS-F and PPM3H-CDS-R generated a 1215 bp amplicon in the wild-type RH strain, but no amplification in the knockout strain (Figure 1D), indicating complete loss of *ppm3h* expression.

In parallel, a gene replacement strain was constructed by introducing RH-derived *ppm3h* into the less virulent PRU background. PCR verification using primers PD-1F/PD-1R and PD-2F/PD-2R (Figures 1E, F), along with HA-tag detection (Figure 1G), confirmed proper integration and expression of the transgene. This gain-of-function model allowed functional assessment of RH-encoded PPM3H in a distinct genetic background and helped differentiate direct effects from broader pleiotropic consequences.

**Figure 2:**
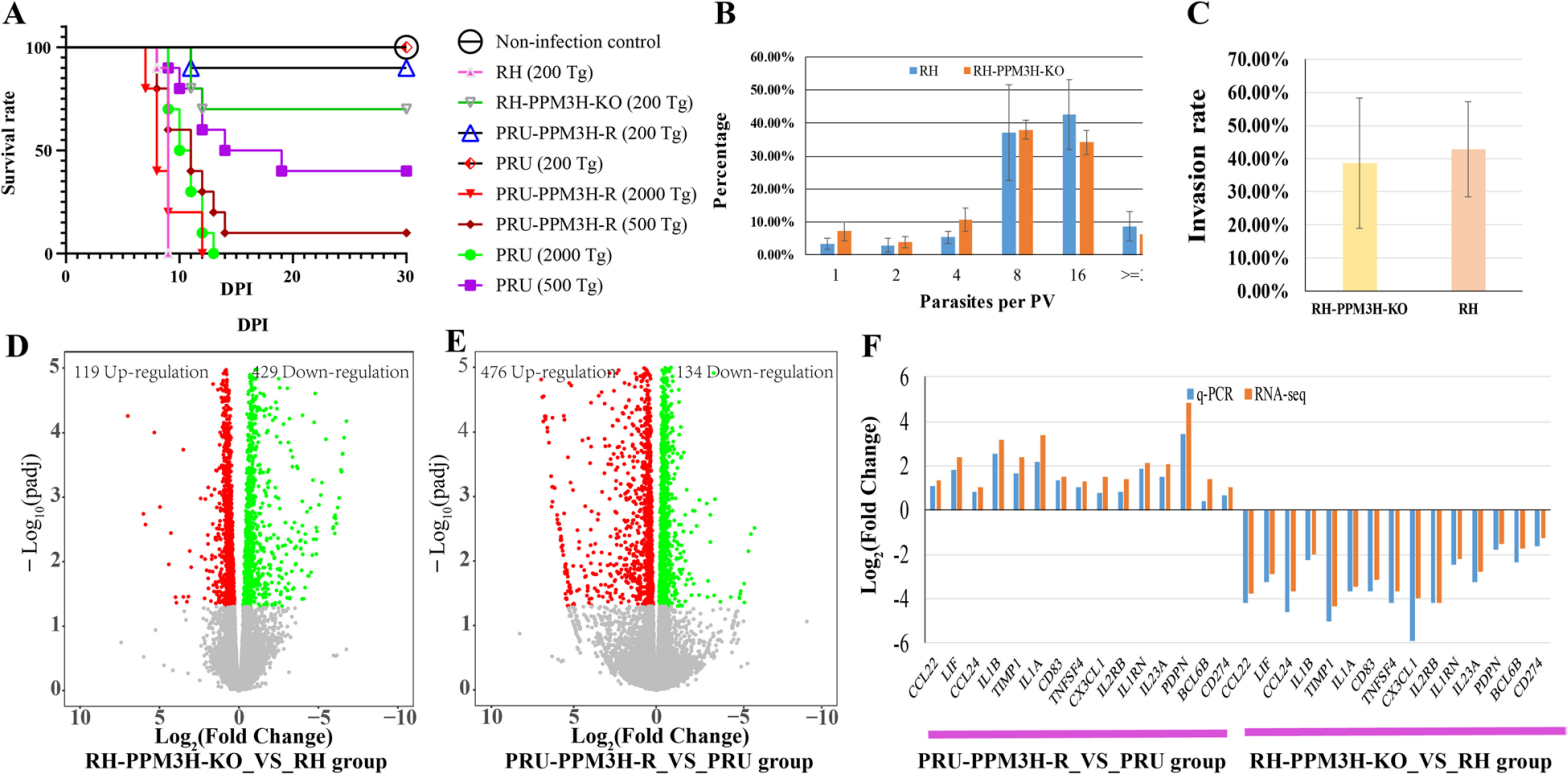
**PPM3H modulates** ***T. gondii* virulence, replication and invasion.** **A** Survival curves of mice infected with varying doses of wild-type and PPM3H-modified *T. gondii* strains (RH and PRU backgrounds). **B** Quantification of intracellular replication in RH wild-type and RH-PPM3H-KO parasites. **C** Host cell invasion assay comparing RH and RH-PPM3H-KO strains. **D-E** Transcriptomic analysis using RNA-seq. Volcano plots show differentially expressed genes (DEGs) in RH-PPM3H-KO vs. RH (**D**) and PRU-PPM3H-R vs. PRU (**E**). **F** qPCR validation of DEGs.

To evaluate the role of PPM3H in *T. gondii* virulence, mice were infected with either the RH-PPM3H-KO or wild-type RH strains. As shown in Figure 2A, 70% of mice infected with the knockout strain survived, whereas all mice infected with the wild-type strain succumbed by day 9 post-infection. These results highlight PPM3H as a key contributor to acute virulence, likely through its involvement in immune evasion, host manipulation, or effector protein regulation essential for in vivo survival.

Further testing in the PRU background showed that expression of RH-derived PPM3H significantly increased lethality compared to wild-type PRU (Figure 2A), demonstrating that PPM3H's virulence-promoting effects are not restricted to the RH genetic context. This transferable gain-of-function phenotype reinforces PPM3H as a conserved virulence determinant and potential therapeutic target.

Interestingly, despite its marked impact on virulence in vivo, deletion of *ppm3h* had no significant effect on parasite replication (Figure 2B) or host cell invasion (Figure 2C) in vitro. The RH and RH-PPM3H-KO strains showed comparable replication rates within PVs and similar invasion efficiency, indicating that PPM3H is dispensable for core parasite fitness traits. This suggests its virulence function operates through more nuanced mechanisms, possibly involving modulation of host–parasite interactions, immune responses, or stress adaptation.

### Impact of *ppm3h *knockout on gene expression in *T. gondii*

As detailed in Additional file 2, knockout of *ppm3h* in the RH strain led to significant changes in the expression of 32 *T. gondii* genes, most of which were upregulated. In contrast, replacing *ppm3h* in the PRU strain with the RH version resulted in differential expression of 38 genes, predominantly downregulated. Importantly, aside from *ppm3h* itself, there were no shared DEGs between the RH-PPM3H-KO and PRU-PPM3H-R strains. The expression level of *ppm3h* in the PRU-PPM3H-R strain was significantly higher than in the wild-type PRU strain (Log₂ Fold Change = 6.48). Additionally, a broad comparison between PRU and RH strains revealed differential expression of 851 *T. gondii* genes, with *ppm3h* expression being substantially higher in RH (Log₂ Fold Change = 2.79), consistent with data from ToxoDB. These findings suggest that *ppm3h* knockout has a limited, yet distinct, influence on *T. gondii* gene expression, while strain-specific regulatory elements likely shape broader expression patterns.

### Gene expression changes in infected RAW264.7 cells and cytokine responses in mice

Infection of RAW264.7 macrophages led to substantial host gene expression changes. In the RH-PPM3H-KO vs. RH comparison, 429 genes were significantly downregulated and 119 upregulated (Figure 2D). In the PRU-PPM3H-R vs. PRU comparison, 610 genes were differentially expressed, with 476 upregulated and 134 downregulated (Figure 2E). These RNA-Seq findings were validated by qPCR analysis of 15 randomly selected mouse genes, confirming the reliability of the dataset (Figure 2F; see Additional file 3).

**Figure 3:**
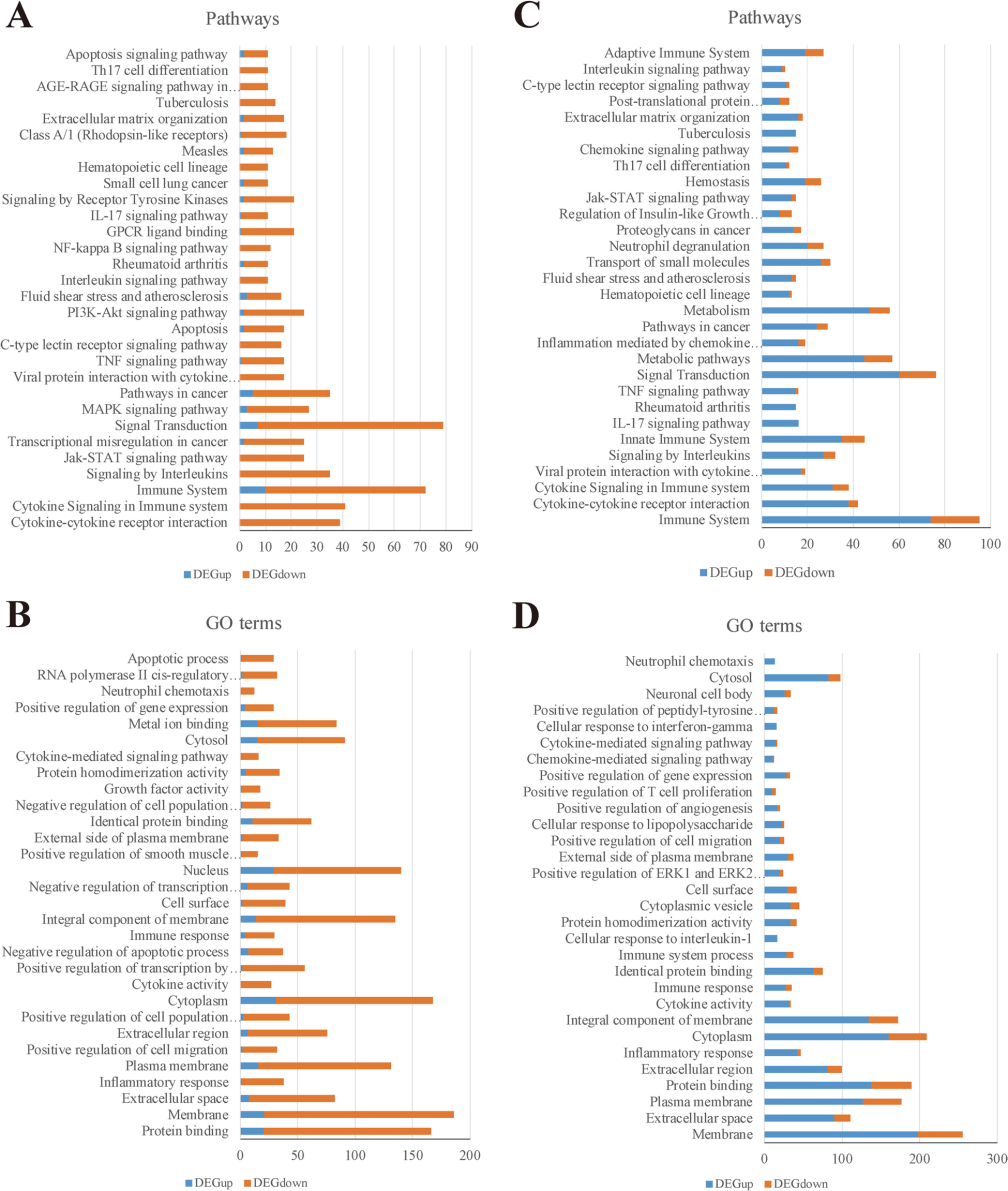
**Pathway and GO enrichment analyses of DEGs in** ***T***. ***gondii*-infected RAW264.7 cells.** **A** Top 30 KEGG pathways among DEGs in RAW264.7 cells infected with RH-PPM3H knockout parasites compared to wild-type RH strain. **B** Top 30 GO terms among DEGs in RAW264.7 cells infected with RH-PPM3H knockout parasites compared to wild-type RH strain. **C** Top 30 enriched pathways from DEGs in PRU-PPM3H reconstituted parasites compared to wild-type PRU. **D** GO term enrichment from the PRU-PPM3H-R vs. PRU comparison.

Pathway and GO enrichment analyses revealed that most of the top 30 enriched pathways in both comparisons were immune-related (Figure 3). These included cytokine-cytokine receptor interaction, Jak-STAT signaling, TNF signaling, IL-17 signaling, Th17 cell differentiation, and neutrophil chemotaxis. In RH-PPM3H-KO vs. RH, immune-related pathways were primarily enriched by downregulated genes (Additional file 4), whereas in PRU-PPM3H-R vs. PRU, they were mainly enriched by upregulated genes (Additional file 5). Serum cytokine analysis in infected mice mirrored these findings, with notable changes in IL-6, IFN-γ, IL-17A, and IL-10 levels (Additional file 6).

**Figure 4:**
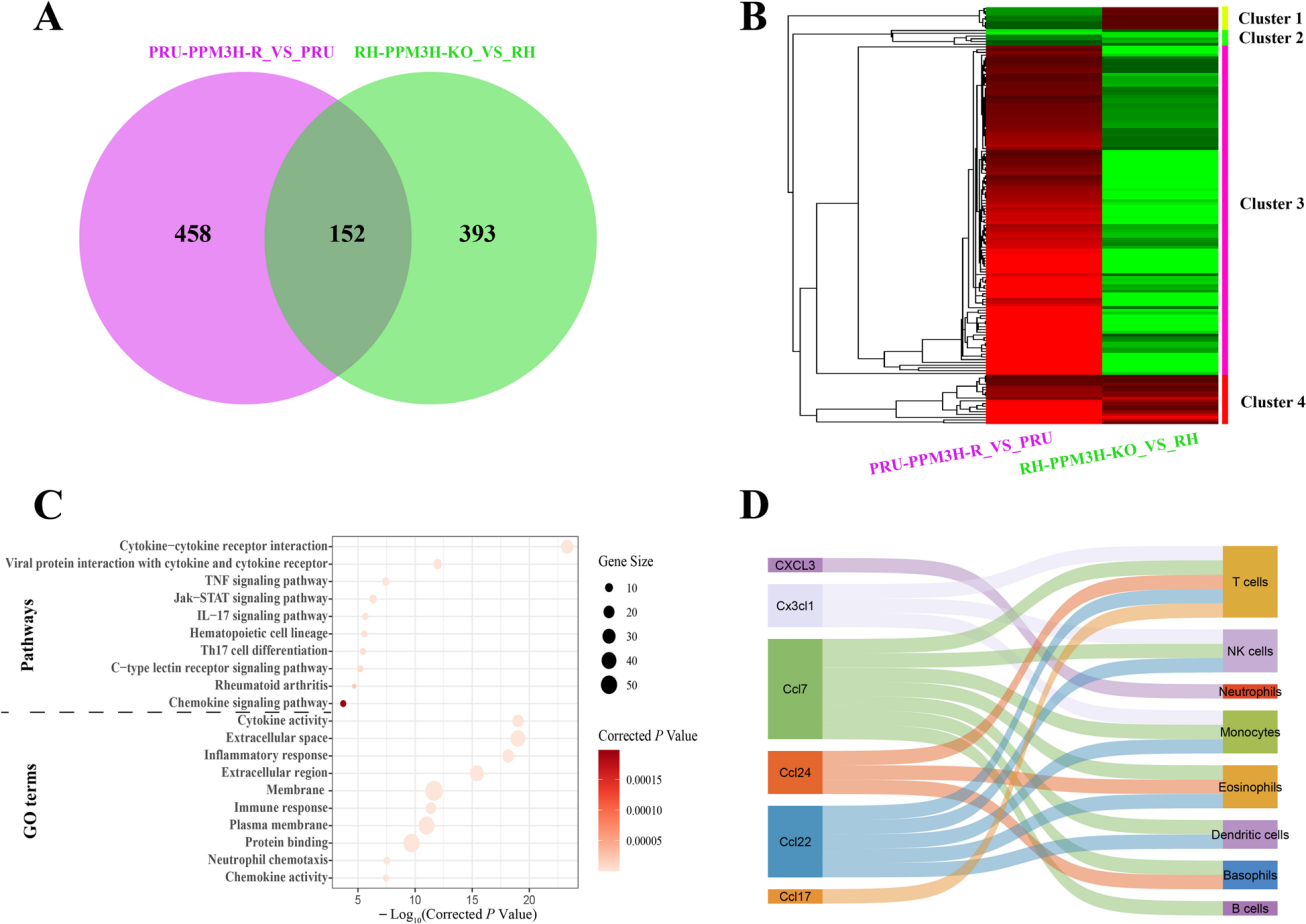
**Comparative transcriptomic analysis of host responses to PPM3H perturbation in two** ***T***. ***gondii*** **strains**. A Venn diagram showing the intersection of DEGs between RH-PPM3H-KO vs. RH and PRU-PPM3H-R vs. PRU groups. A substantial overlap (152 genes) indicates shared host responses to PPM3H expression changes across strains. **B** Heatmap of the 152 shared DEGs clustered into expression modules. Cluster 1 and Cluster 3 display opposing expression trends, potentially reflecting divergent pathways activated or suppressed by PPM3H. **C** Top 10 enriched pathways and GO terms identified from the DEGs in clusters 1 and 3. **D** Chemokine interaction network derived from shared DEGs. Node annotations reflect inferred cellular origins (e.g., dendritic cells, eosinophils).

We identified 152 DEGs common to both the RH-PPM3H-KO vs. RH and PRU-PPM3H-R vs. PRU comparisons (Figure 4A). These were grouped into four expression clusters: Clusters 2 (6 genes) and 4 (18 genes) showed consistent expression patterns across both models, while Clusters 1 (8 genes) and 3 (120 genes) displayed opposing trends (Figure 4B; Additional file 7). Enrichment analysis of Clusters 1 and 3 revealed significant involvement in inflammatory and immune pathways (27 pathways and 139 GO terms; Figure 4C, Additional file 8). Importantly, six chemokines—Ccl17, Ccl22, Ccl24, Ccl7, Cx3cl1, and Cxcl3—were identified within Cluster 3 and associated with chemotaxis of eight immune cell types (Figure 4D; Additional file 9), implicating PPM3H in the modulation of immune cell recruitment. These findings collectively indicate that PPM3H modulates host immune signaling by broadly impacting transcriptional networks, particularly those governing cytokine and chemokine responses. This regulation shapes a conserved chemokine-driven immune signature, potentially influencing immune evasion and cell recruitment strategies across different *T. gondii* strains and contributing to variations in parasite virulence and host–pathogen interactions.

### Co-expression analysis of host and *T. gondii* genes with *ppm3h*

**Figure 5:**
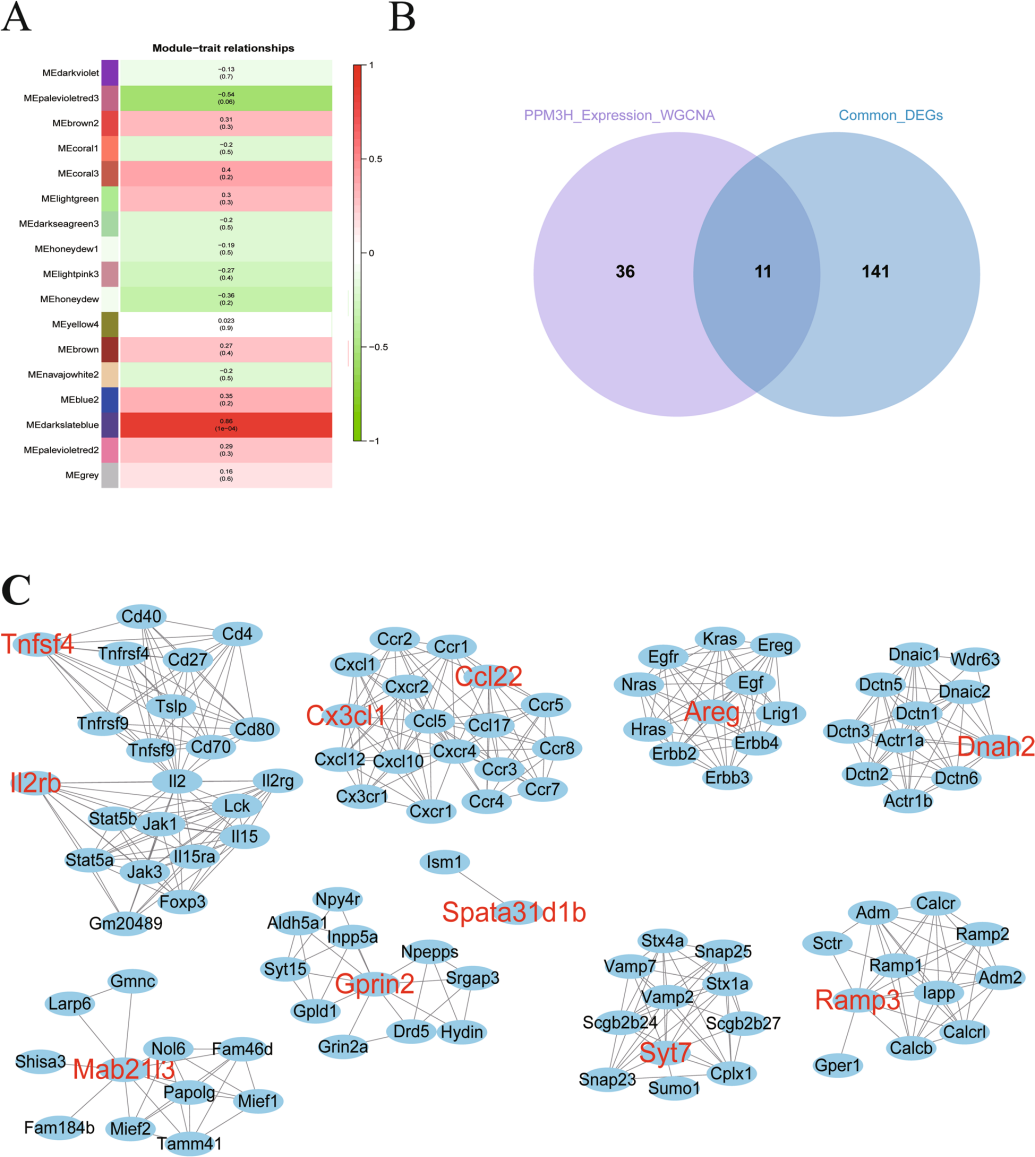
**Host genes and interaction networks significantly associated with *T***. ***gondii*** ***ppm3h*** **expression. A** Correlation matrix of gene co-expression modules with *ppm3h* expression using WGCNA. The MEpalevioletred2 module shows the strongest positive correlation (*r* = 0.86, *p* = 1e − 4), suggesting it may contain critical *ppm3h*-responsive host gene. **B** Identification of 11 host genes common across significant DEGs and modules correlated with *ppm3h*. These genes may serve as key markers or effectors of PPM3H-mediated host modulation. **C** Protein–protein interaction network of these 11 genes, constructed using known interactions. Genes significantly associated with *ppm3h* are labeled in red, while interacting partners are labeled in black.

WGCNA was used to identify host genes co-expressed with *T. gondii ppm3h*. Among 17 mouse gene modules, the MEdarkslateblue module showed significant correlation (Figure 5A), with 47 genes exhibiting a gene significance *P*-value < 0.01. Key genes included *Cx3cl1*, *Ccl22*, *Tnfsf4*, *Il2rb*, *Il12rb2*, and *Csf2rb2*. Pathway analysis revealed strong enrichment in immune-related pathways, particularly cytokine-cytokine receptor interaction (FDR = 9.71E-06) and viral protein interaction with cytokines (FDR = 0.0007).

Intersection analysis revealed 11 host genes among the 47 that were differentially expressed in both *ppm3h*-modified infection models (Figure 5B; Additional file 9). Protein interaction network analysis identified 97 interacting partners (Figure 5C), with enrichment in immune pathways including Jak-STAT, chemokine signaling, Th17 differentiation, and the IgA immune network (Additional file 10). These analyses suggest specific host transcriptional networks and interaction hubs influenced by *ppm3h*, supporting its role in modulating immune-related processes at a systems level and identifying potential targets for further functional validation.

**Figure 6:**
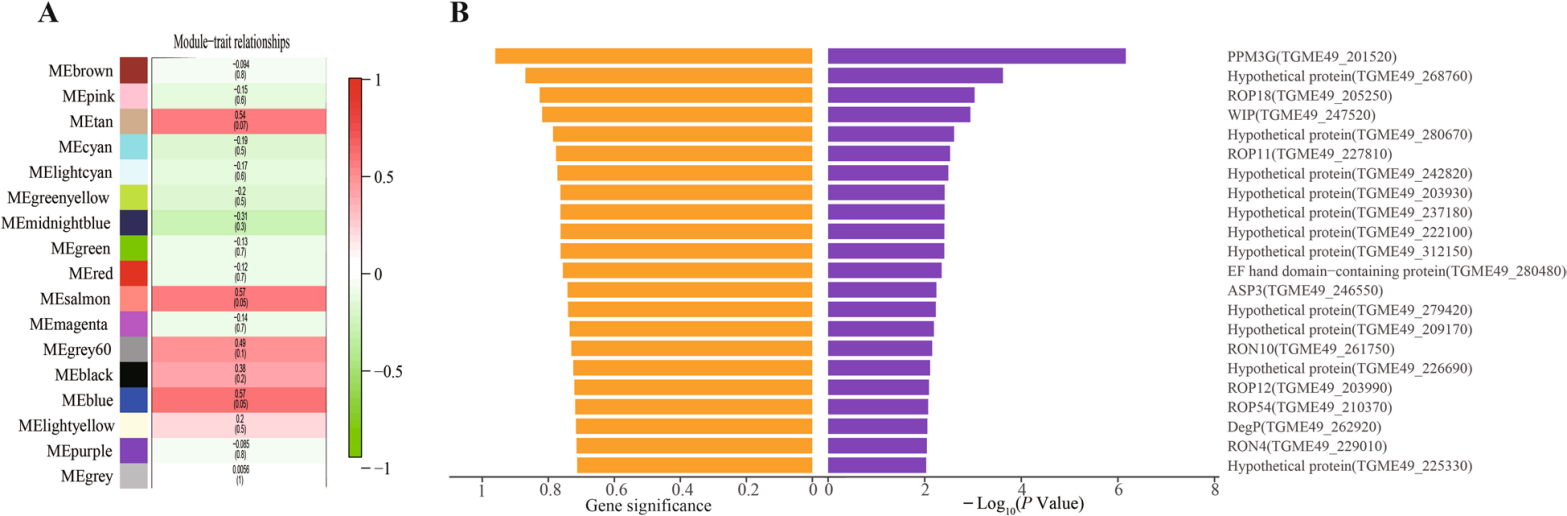
***Toxoplasma gondii* genes significantly correlated with** ***ppm3h* expression.** **A** Module-trait correlation matrix derived from WGCNA. The salmon module shows the strongest positive correlation with *ppm3h* expression (*r* = 0.57, *p* = 0.05), suggesting coordinated transcriptional regulation. **B** List of individual *T. gondii* genes significantly co-expressed with *ppm3h*, with enrichment for rhoptry proteins.

WGCNA was also applied to *T. gondii* genes, revealing 17 modules, with MEsalmon and MEblue showing strong co-expression with *ppm3h* (Figure 6A). A total of 541 *T. gondii* genes were significantly co-expressed (gene significance *p* < 0.01), many of which encode hypothetical proteins (Additional file 11). Interestingly, 22 rhoptry protein coding genes were among the co-expressed genes, including *rop18*, *rop11*, *rop12*, *rop23*, *rop46*, *rop54*, *ron4*, and *ron10*, as well as effector proteins such as ASP3 and DegP (Figure 6B). Given ROP18’s known role in modulating host immunity, these findings support a functional link between PPM3H and parasite-driven immune regulation.

## Discussion

*T. gondii* is one the most widespread and successful intracellular pathogens, capable of infecting virtually all warm-blooded vertebrates [30]. In mice, infection with a virulent strain such as RH is lethal, while attenuated strains such as PRU cause minor pathology, allowing its survival. Numerous virulence factors have been identified, including rhoptry protein kinases ROP16 [6] and ROP18 [13], which manipulate host immune signaling via protein phosphorylation, targeting pathways such as STAT3 [6] and NF-κB [31]. While kinases promote phosphorylation, phosphatases counterbalance this by dephosphorylating target proteins. Phosphatases are recognized as virulence regulators in pathogens such as *Francisella* [19, 32], *Mycobacterium* [33] and *Plasmodium* [20]; however their roles in *T. gondii* remain poorly characterized. Among *T. gondii* phosphatases, PPM3H has emerged as a novel candidate that appears to modulate host immune responses rather than directly affecting parasite biology. Nevertheless, its contribution to *T. gondii* virulence in vivo has been unclear.

To investigate this, we engineered two strains: an RH strain with *ppm3h* deleted (RH-PPM3H-KO) and a PRU strain expressing the RH allele of *ppm3h* including its regulatory elements (PRU-PPM3H-R). Virulence assays showed that deleting *ppm3h* from RH significantly improved host survival, whereas introducing the RH-derived *ppm3h* into PRU markedly reduced mouse survival. These virulence changes occurred without affecting parasite replication, host cell invasion, or global gene expression, indicating that PPM3H modulates virulence primarily through altering host–pathogen interactions rather than intrinsic parasite fitness. Interestingly, according to our sequencing of *ppm3h* CDS, the *ppm3h* coding sequence is identical in RH and PRU, suggesting that virulence differences arise from regulatory variation. Transcriptomic and ToxoDB data confirmed higher *ppm3h* expression in type I strains (e.g., RH) compared to type II strains (e.g., PRU). By replacing the promoter and 3' UTR in PRU with those from RH, we elevated *ppm3h* expression to mimic the high-expression phenotype linked to virulence. This strategy, keeping the coding sequence constant while altering regulatory control, allowed us to isolate the effects of increased expression on virulence, with PRU’s lower baseline pathogenicity providing an ideal model to study RH-specific virulence dynamics.

Our transcriptomic analysis confirmed significantly higher *ppm3h* expression in RH compared to PRU (Log_2_ Fold Change = 2.79), and even greater levels in PRU-PPM3H-R (Log_2_ Fold Change = 6.48) (Additional file 2). Sequence comparisons revealed multiple SNPs and InDels in regulatory regions likely underlying this differential expression. Elevated *ppm3h* expression correlated with enhanced inflammatory responses in infected mice, including increased serum IL-6, IL-17A, and IL-10, alongside decreased IFN-γ, consistent with transcriptional profiles of RAW264.7 macrophages infected with PRU-PPM3H-R. Although PPM3H has minimal direct effects on the parasite, it profoundly influences host gene expression. In macrophages infected with PRU-PPM3H-R versus PRU, 610 host genes were differentially expressed (476 upregulated, 134 downregulated). Conversely, in RH-PPM3H-KO versus RH, 429 genes were downregulated and 119 upregulated (Additional file 3, Figures 2D, E). GO and pathway enrichment analyses revealed these genes to be predominantly involved in immune and inflammatory responses. Importantly, most inflammation- and immune-related genes were downregulated in the absence of *ppm3h* (RH-PPM3H-KO), suggesting PPM3H promotes host inflammatory gene expression (Figures 3A, B).

Because excessive inflammation can cause severe immunopathology in hosts infected with *T. gondii* [34], we performed an intersection analysis of DEGs regulated by *ppm3h*. Among 152 shared DEGs between PRU-PPM3H-R vs. PRU and RH-PPM3H-KO vs. RH (Figure 4A), four expression clusters were identified. Cluster 2 (6 genes) and Cluster 4 (18 genes) exhibited consistent expression trends, while Cluster 1 (8 genes) and Cluster 3 (120 genes) showed opposite regulation trends in the two comparisons, consistent with *ppm3h*-dependent control (Figure 4B). These clusters were further analyzed for functional enrichment, revealing significant overrepresentation of immune and inflammatory pathways (Additional file 8). Key pathways included Th17 cell differentiation, Jak-STAT signaling, cytokine–cytokine receptor interaction, and chemokine-mediated signaling, pathways central to cytokine storm induction, a fatal immune dysregulation outcome during *T. gondii* infection [34, 35].

Among the 152 common DEGs, six cytokine storm biomarkers (*Tnfrsf1b*, *Tnfsf4*, *Tnfrsf8*, *Tnfsf15*, *Il6*, and *Il33*) were all upregulated in Cluster 3, reinforcing PPM3H’s role as a pro-inflammatory factor driving cytokine storm–like responses. Since cytokine storms and immunopathology are largely mediated by chemokines [35], we identified six chemokine genes (*Ccl17*, *Ccl22*, *Ccl24*, *Ccl7*, *Cx3cl1*, and *Cxcl3*) among shared DEGs in Clusters 1 and 3. These chemokines are known to recruit eight key immune or immunopathological cell types, including T cells, dendritic cells, eosinophils, natural killer (NK) cells, monocytes, basophils, B cells, and neutrophils [36, 37] (Figure 4D). Interestingly, T cells, monocytes, and NK cells were each targeted by more than three of these chemokines. Although these immune cells play essential roles in controlling infections, their excessive recruitment can cause severe pathology in infected hosts [35]. Among the identified chemokines, *Ccl7* stands out for its ability to attract all eight cell types, and its overexpression is linked to increased host susceptibility to *T. gondii* [38]. This suggests that PPM3H may enhance parasite virulence by upregulating chemokine expression, promoting excessive immune cell infiltration and immunopathology.

To further explore host gene networks associated with *ppm3h* expression, we performed WGCNA. Mouse genes clustered into 17 distinct expression modules, with the MEdarkslateblue module showing significant correlation with *ppm3h*. Within this module, 47 genes were co-expressed with *ppm3h* and enriched in immune- and inflammation-related pathways, such as cytokine–cytokine receptor interaction and viral protein–cytokine receptor interaction. Eleven mouse genes (*Areg*, *Ccl22*, *Cx3cl1*, *Dnah2*, *Gprin2*, *Il2rb*, *Mab21l3*, *Ramp3*, *Spata31d1b*, *Syt7*, and *Tnfsf4*) were both co-expressed with *ppm3h* and differentially expressed in PRU-PPM3H-R vs. PRU and RH-PPM3H-KO vs. RH comparisons (Figure 5B), all belonging to Cluster 3 (Additional file 9). Several, including *Areg*, *Il2rb*, and *Tnfsf4*, are key immune and inflammatory regulators [39–41], with *Il2rb* and *Tnfsf4* (*Ox40l*) implicated in cytokine storm development. *Syt7* participates in calcium-regulated lysosome exocytosis [42], while *Ccl22* and *Cx3cl1* mediate chemotaxis of immune cells such as T cells, NK cells, monocytes, eosinophils, and dendritic cells [36, 37]. Using STRING, 97 proteins interacting with these 11 genes were identified (Figure 5C), many involved in inflammation and immune pathways (Additional file 10). Together, these findings support a model in which PPM3H promotes parasite virulence by enhancing cytokine- and chemokine-driven inflammation that can lead to lethal immunopathology.

Genes with similar expression patterns often share functions [43–48]. Therefore, identifying *T. gondii* genes co-expressed with *ppm3h* may illuminate how it regulates host responses and reveals broader parasite virulence strategies. We grouped *T. gondii* genes into 17 modules; among these, MEsalmon and MEblue showed strong correlations with *ppm3h*, containing 541 co-expressed genes (Additional file 11). Focusing on rhoptry protein coding genes, 22 rhoptry gene members, including established virulence factor genes *rop18* and *rop54*, co-expressed with *ppm3h* (Figure 6B). ROP18 and ROP54 are serine/threonine kinases that facilitate immune evasion by phosphorylating host IRGs and GBPs. In contrast, PPM3H as a phosphatase, dephosphorylates host substrates to enhance inflammatory and immune responses, contributing to *T. gondii* pathogenicity in mice. This suggests a novel virulence mechanism whereby *T. gondii* coordinates kinase and phosphatase activities (e.g., ROP18, ROP54, PPM3H) to finely modulate host immunity, either suppressing defences or promoting harmful immune activation.

Although PPM3H is confirmed as a rhoptry protein identified in proteomic datasets and clustered with canonical rhoptry kinases such as ROP18 and ROP54, its secretion into host cells and subcellular localization remain unverified. These are critical knowledge gaps, and future studies using the PRU-PPM3H-3HA strain should utilize immunofluorescence assays to clarify PPM3H’s secretion and intracellular localization. We propose three mechanisms through which PPM3H may modulate host gene expression and contribute to *T. gondii* virulence. First, PPM3H could act as a secreted phosphatase that directly dephosphorylates specific host or parasite substrates, thereby enhancing inflammatory responses. Second, PPM3H may function as a maturase, similar to ASP3 [49], regulating the maturation or activation of other parasite effectors, including kinases like ROP18 and ROP54, thus indirectly modulating host immunity. Third, PPM3H may influence the export of effector proteins into host cells, akin to the role described for PPM3C [23], altering the parasite′s ability to deliver key virulence factors. These mechanisms suggest that PPM3H might also operate upstream in parasite signalling networks, coordinating phosphorylation and dephosphorylation cascades that collectively shape virulence. Together, these hypotheses offer a robust framework for future investigation into the molecular function of PPM3H in *T. gondii* pathogenesis.

In conclusion, our findings confirm that PPM3H contributes to the virulence of *T. gondii*. Transcriptomic analyses revealed that although PPM3H has minimal effect on parasite gene expression, it markedly alters host inflammatory responses. Unlike many virulence factors that promote immune evasion and suppress host immunity to aid parasite survival, PPM3H exacerbates immune activation, leading to lethal immunopathology. These findings broaden our understanding of *T. gondii* virulence by highlighting a novel pathogenic mechanism and revealing a potential target for therapeutic intervention.

## Supplementary Information

**Additional file 1. List of primers used in this study.**

**Additional file 2. Differentially expressed** ***T***. ***gondii*** **genes across the RH-PPM3H-KO vs. RH, PRU-PPM3H-R vs. PRU, and PRU vs. RH comparisons.** The Log_2_ (Fold Change) column indicates the magnitude of gene expression differences between groups. The *P* value column presents the Wald test results; the padj column shows the Benjamini–Hochberg–adjusted *P* values, and the description column provides gene names or functional annotations.

**Additional file 3.** **Differentially expressed mouse genes in the RH-PPM3H-KO vs. RH and PRU-PPM3H-R vs. PRU comparisons.** The Log_2_ (Fold Change) column reflects the extent of gene expression changes between groups; the*P* value column shows results from the Wald test; the padj column reports Benjamini–Hochberg–adjusted *P* values; and the description column lists gene names or functional annotations.

**Additional file 4.** **Significantly enriched pathways and GO terms among DEGs in RAW264.7 cells from the RH-PPM3H-KO vs. RH comparison.**

**Additional file 5.** **Significantly enriched pathways and GO terms among DEGs in RAW264.7 cells from the PRU-PPM3H-R vs. PRU comparison.**

**Additional file 6.** **Cytokine profiles in mice infected with the PRU or PRU-PPM3H-R strains.** The green bars represent the Log₂ (Fold Change) in serum cytokine levels between nine surviving mice infected with 200 PRU tachyzoites and nine surviving mice infected with 200 PRU-PPM3H-R tachyzoites. The orange bars indicate the Log₂ (Fold Change) in gene expression in RAW264.7 cells infected with the PRU strain versus those infected with the PRU-PPM3H-R strain.

**Additional file 7.** **List of the 152 mouse DEGs shared between the RH-PPM3H-KO vs. RH and PRU-PPM3H-R vs. PRU comparisons.**

**Additional file 8.** **Pathway and GO enrichment analyses of mouse genes in Cluster 1 and Cluster 3.**

**Additional file 9. Differentially expressed mouse genes associated with** ***T***. ***gondii***  ***p expression.pm3h***

**Additional file 10. Pathway and GO enrichment analyses of proteins interacting with common DEGs significantly linked to** ***T***. ***gondii*** ***ppm3h*** **expression.**

**Additional file 11. Details of** ***T***. ***gondii*** **genes co-expressed with** ***ppm3h. Subcellular localization information was obtained from the ToxoDB database (Version: ToxoDB-60_TgondiiME49).pm3h***

## Data Availability

All data generated or analyzed during this study are included in this published article. RNA sequencing datasets have been deposited in the NCBI SRA database under the accession number PRJNA1010983. Supplementary material associated with this article can be found in the online version.
