# Supplementary figures and images for: Toxoplasma gondii PPM3H regulates the parasite virulence and modulates host immune and inflammatory responses in mice
Source: Vet Res. 2025 Aug 26;56:171. doi: 10.1186/s13567-025-01603-y (PMC12379425; doi:10.1186/s13567-025-01603-y)

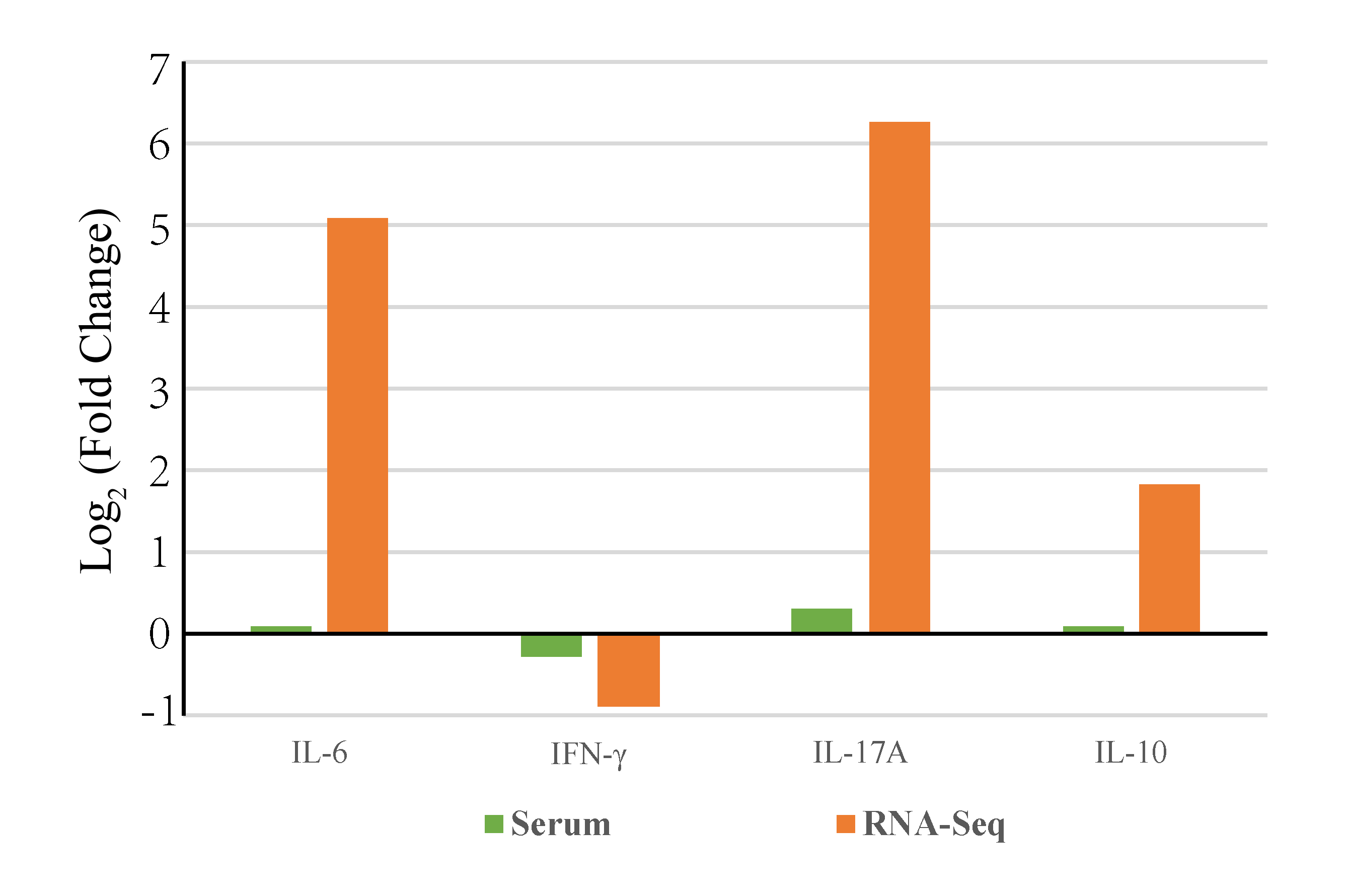

Supplement: Supplementary file 6 — Additional file 6. Cytokine profiles in mice infected with the PRU or PRU-PPM3H-R strains. The green bars represent the Log₂ (Fold Change) in serum cytokine levels between nine surviving mice infected with 200 PRU tachyzoites and nine surviving mice infected with 200 PRU-PPM3H-R tachyzoites. The orange bars indicate the Log₂ (Fold Change) in gene expression in RAW264.7 cells infected with the PRU strain versus those infected with the PRU-PPM3H-R strain. [file 13567_2025_1603_MOESM6_ESM.tiff]
